# Minimally invasive mini-hemilaminectomy-corpectomy in cadaveric dogs: evaluation of the accuracy and safety of a three-dimensionally printed patient-specific surgical guide

**DOI:** 10.1186/s12917-022-03374-6

**Published:** 2022-07-13

**Authors:** Jinsu Kang, Seungeon Lee, Namsoo Kim, Suyoung Heo

**Affiliations:** Institutional Address: College of Veterinary Medicine, Jeonbuk National University, Gobong-ro 79, Iksan, 54596 Republic of Korea

**Keywords:** Minimally invasive spine surgery, Three-dimensional, Patient-specific guide, Endoscopic system, Dog

## Abstract

**Background:**

As the frequency of spine surgery increases in the veterinary field, many studies have been conducted on minimally invasive spine surgery (MISS). Although many studies have been conducted on the thoracolumbar spine about MISS in animals, several problems and limitations have emerged regarding this method. Therefore, we developed a three-dimensional (3D) printed patient-specific surgical guide (3DPSSG) using 3D printing technology to overcome these problems. We aimed to evaluate the accuracy and safety of the 3DPSSG in minimally invasive mini-hemilaminectomy-corpectomy (MI-MHC).

MI-MHC using 3DPSSG and an endoscopic system was performed at L1–L2 in 15 cadaveric dogs. The procedure of fixing the surgical guide to the vertebral body through screws and the surgical procedure using the guide were performed by two surgeons with different experiences. Postoperative computed tomography was used to measure planned and postoperative screw trajectories (angle, protruding from the far cortex) and to create 3D rendering images of vertebrae to evaluate the direction of bone window formation, corpectomy slot length, depth, and height ratio.

**Results:**

The two groups which performed by two surgeons with different experiences did not differ in terms of screw angle deviation and length of the screw protruded from the far cortex. The corpectomy slot-length ratio was not different between the two groups; however, the slot-depth and height ratios were different.

**Conclusions:**

No differences were detected in screw trajectory and corpectomy slot-length ratio between the two groups. The 3DPSSG for MI-MHC is classified as accurate and safe; therefore, it can be an alternative to the conventional technique in dogs.

## Background

Intervertebral disc disease (IVDD) is considered the most common cause of spinal cord trauma, and thoracolumbar lesions account for 66%–87% of all IVDD lesions in dogs [1–3]. Until recently, hemilaminectomy has been widely performed as a standard treatment for spinal cord decompression, but a certain degree of spinal cord manipulation is frequently inevitable during surgery, and deterioration of neurological symptoms after surgery has also been observed [4–8].

Mini-hemilaminectomy was first introduced by Braund in 1976; it is a surgical method that allows one access to the spinal canal while removing the accessory process and small amount of bone tissue during widening the intervertebral foramen cranially and caudally [4, 9]. Articular facets are preserved, and especially when combined with fenestration, vertebral column stability is maintained better than with hemilaminectomy [4, 10, 11]. During mini-hemilaminectomy, it is easier to remove disc material located in the close to the ventral part of the spinal cord, reducing the risk of iatrogenic spinal cord trauma and the formation of laminar membranes [12]. Mini-hemilaminectomy has been shown to have better prognosis in terms of postoperative condition and recovery time than hemilaminectomy in small and large dogs [4, 13–15]. In addition, partial lateral corpectomy has recently been used to reduce surgical manipulation of the spinal cord by removing portions of the vertebral body adjacent to the intervertebral disc and addressing herniated and compressing disc material in the ventral direction [9, 16, 17].

Currently, as the frequency of spine surgery increases in the veterinary field, many studies are being conducted on minimally invasive spine surgery (MISS), which is performed with minimal damage to the skin, muscles, and other surrounding soft tissues [18–28].

The methods to access the vertebral column for MISS include the percutaneous approach and the use of a tubular or expendable retractor. The percutaneous approach is mainly used to treat small lesions. Illumination and magnification are performed using an endoscope, and correct placement is confirmed using intraoperative fluoroscopy. Access through a tubular or expandable retractor is necessary when a large surgical area is required. Illumination and magnification are most commonly performed using an operating microscope, and correct placement is verified using intraoperative fluoroscopy [25].

Although many studies have been conducted on the thoracolumbar spine in animals, several problems and limitations have emerged regarding MISS [19, 21–23, 26–28]. First, preservation of the articular process and its tendinous attachments through conversion to mini-hemilaminectomy is best suited to the basic principle of MISS [25]. Second, when a cannula is installed and surgery is performed using an endoscope, the cannula may be shifted in the caudal direction during the operation because of the caudal slope of the articular process [27]. Additionally, because the blunt edge of the cannula is on an uneven bone surface, the muscles or surrounding tissues may invade the visual field. In this case, repositioning of the cannula is required, which requires refitting the smaller dilation sleeves [27, 28].

Therefore, in this study, we aimed to produce a three-dimensional (3D) printed patient-specific surgical guide (3DPSSG) using 3D printing technology to overcome these limitations. To the best of our knowledge, at the time of writing, there are no published reports or studies describing the use of 3DPSSG for minimally invasive mini-hemilaminectomy-corpectomy (MI-MHC) in dogs. This study aimed to evaluate the accuracy and safety of the 3DPSSG for MI-MHC in cadaveric dogs.

## Results

### Cadaveric data

Fifteen cadaveric dogs met the inclusion criteria. Seven were Mongrel dogs, three were Jindo dogs, two were Shih tzus, two were Pomeranians, and one was a Maltese. Seven were female and eight were male. The median body weight of the cadavers was 10.25 ± 3.31 kg.

### Design and manufacture of the surgical guide

Various prototypes were designed and printed to determine the size of the drill bit, the size and length of the screw, and to facilitate drilling and screw installation. Fifteen surgical guides were created, and the mean design time was 29.31 ± 1.32 min.

### Total surgical time

The average total surgical time consisting of guide installation, surgical procedure in fifteen specimens was 77.8 ± 7.87 min.

### Postoperative evaluation: surgical guide accuracy

Thirty screws were inserted into 30 vertebral bodies (15 screws in L1 and 15 screws in L2). The screw angle deviation was 2.032° ± 0.531 in Group A (screws inserted by a surgeon with seven years of experience) and 2.151° ± 0.326 in Group B (screws inserted by a surgeon with two years of experience) (*p* = 0.367). The length of the screw protruding from the far cortex of the vertebrae was 0.513 mm ± 0.102 in Group A and 0.509 mm ± 0.089 in Group B (*p* = 0.461). There were no differences in the values between Groups A and B (Table 1).

**Table 1:** Deviations of the screw angle, length of the screw protruding from the far cortex for experienced group (Group A) and inexperienced group (Group B)

Measurement	Group A (*n* = 15)	Group B (*n* = 15)	*P*-value
Screw angle deviation, °	2.032 ± 0.531	2.151 ± 0.326	.367
Length of the screw protruding from the far cortex, mm	0.513 ± 0.102	0.509 ± 0.089	.461

### Postoperative evaluation: screw placement safety

To evaluate the safety of the surgery using the guide, 15 cadavers and 30 screws (15 screws in L1 and 15 screws in L2) were investigated. Screw placement was evaluated using a modified Zdichavsky classification system [29]. Of the 28 screws that were classified as Grade I, two screws were classified as Grade IIa. No different grades of classification were observed in the present study.

### Postoperative evaluation: direction of bone window formation

The value of the directional bias assessment was greater than one in seven cases (1.215 ± 0.095), and bias was evaluated in the cranial direction. The value was less than one in eight cases (0.849 ± 0.087), and bias was evaluated in the caudal direction.

### Postoperative evaluation: corpectomy slot formation

To evaluate the safety of the surgery using the guide, a total of 15 slots and 30 Sects. (15 sections in L1 and 15 sections in L2) were investigated. In Group C, the corpectomy slot-length ratio was 22.29 ± 2.64 in Group C and 20.35 ± 4.50 in Group D (*p* = 0.567). The corpectomy slot-depth ratio was 30.13 ± 3.53 in Group C and 23.29 ± 4.06 in Group D (*p* = 0.001). The corpectomy slot-height ratio was 32.47 ± 2.91 in Group C and 29.43 ± 5.38 in Group D (p = 0.047) (Table 2).

**Table 2:** Corpectomy slot length, depth, height ratio for experienced group (Group C) and inexperienced group (Group D)

Measurement	Group C	Group D	*P*-value
Corpectomy slot length ratio, %	22.29 ± 2.64	20.35 ± 4.50	.567
Corpectomy slot depth ratio, %	30.13 ± 3.53	23.29 ± 4.06	.001
Corpectomy slot height ratio, %	32.47 ± 2.91	29.43 ± 5.38	.047

## Discussion

In the present study, we demonstrated the accuracy and safety of the 3DPSSG for MI-MHC in cadaveric dogs. Through postoperative CT images and 3D modelling, the accuracy of screw placement required to implant the guide was confirmed. The degrees of deviation from the planned screw angle were small, and the screw placements in this study were graded as Zdichavsky Grade I in 28 cases, except for two cases, and showed an optimal placement rate with a 93.3% probability. In these two cases, there was no case of full penetration of the medial pedicle or invasion of the spinal canal.

The lengths of the screws protruded from the far cortex were measured to be more than 0.5 mm as planned, except in two cases, and these two cases were measured to be less than 1 mm in length, which was evaluated to be within a range that did not damage visceral organs or surrounding soft tissues. The installation of screws did not differ when used by experienced surgeons compared with inexperienced surgeons, providing evidence that the use of the 3DPSSG for MI-MHC is relatively easy to learn and can be applied well, regardless of surgical experience.

The corpectomy slot-length ratio did not differ between the experienced and inexperienced surgeons (*p* = 0.567). However, the slot-depth ratio (*p* = 0.001) and slot-height ratio (*p* = 0.047) of corpectomy differed between experienced and inexperienced operators. This means that the guide can aid in determining the length of the corpectomy by securing the visual field; however, it cannot provide accurate indications regarding the slot depth and height of corpectomy. Therefore, we evaluated whether the corpectomy procedure was influenced by surgical experience.

In the evaluation of whether the bone window of mini-hemilaminectomy is formed by bias in either the cranial or caudal direction, no bias in any specific direction was observed. In a previous study of hemilaminectomy performed with a cannula in dogs, it was found that, relative to the centre of the intervertebral disc space, the length of the exposed spinal canal was significantly longer in the caudal direction than in the cranial direction. These results were presumed to be affected by the position where the cannula was initially placed; in particular, it was presumed that the cannula shifted caudally during the procedure owing to the caudal slope of the articular process [27]. Reflecting this, this guide was designed to be firmly fixed by insertion into the articular process. Through this, it was possible to prevent the guide from shifting in a specific direction during the procedure, as well as to prevent the extent of the bone window from expanding in a specific direction with respect to the intervertebral disc space.

Since preservation of the articular process and attaching soft tissues in mini-hemilaminectomy is the most appropriate principle for the MISS approach [25], soft tissue damage to the area except the periforaminal area was minimised using dilators customised for dogs. The part where the dilator contacts the soft tissue has a sharp shape when using the fillet function of the design software (Fusion 360); this was designed to be suitable for intramuscular approaches, but it was made to have atraumatic characteristics.

In this study, the mean total surgical time was 77.8 ± 7.87 min. Since there are few reports on the surgical time of lateral corpectomy in the veterinary literature, it is difficult to directly compare the MI-MHC time in this study with other studies. In addition, since it was a study through cadaver, the time that could increase due to minor complications such as bleeding was not reflected. However, in a previous experiment of mini-hemilaminectomy using dilators, a cannula system (Medtronic, Minneapolis, MN, USA), and an endoscope system (Richard wolf GmbH, Knittlingen, Germany) performed by the author of this paper, the average operation time was 90.53 ± 8.97 min, excluding the corpectomy procedure.

The reduced total surgical time with this guide can be attributed to several factors. First, the soft tissues and muscles around the periforaminal area were completely separated using the guide. Because the blunt edge of a conventional tubular retractor is on an uneven bone surface, residual muscle and soft tissue are often present between the tip of the tubular retractor and the bone [28]. However, except for at the space where the nerve root passes through, this guide was designed to be compressed in accordance with the shape of the bone surface. After fixing the guide to the vertebral body, if only the soft tissues and muscles remaining inside were removed, the visual field could be secured without obstruction during the procedure. Therefore, there is no need to secure the visual field by removing the soft tissue or muscle that enters the space between the guide and bone surface; thus, the surgical time can be reduced. Second, the guide does not require repositioning. In a previous study on hemilaminectomy using a cannula, if the visual field was obstructed by the invasion of muscle or other soft tissue or by shifting of the cannula during the procedure, it was necessary to start again with a small dilation sleeve in order to install the cannula [27]. In cases where the surgical site is not moved, this repositioning process is not required; thus, the surgical time can be reduced. Finally, intraoperative fluoroscopy was used only to evaluate whether the surgical position was accurate and whether the guide was installed correctly. Through this, radiation exposure and time of fluoroscopic evaluation were also reduced.

In previous studies, the use of a Gelpi retractor rather than a tubular retractor was recommended for small dogs with small paraspinal muscle masses [30]. In this study, MI-MHC was performed by manufacturing dilators and guides suitable for each dog, regardless of their size, and the procedure could be performed without much difficulty, even in small dogs. In addition, since it was customised to fit each dog, the size of the guide was designed to create a bone window and corpectomy slot according to the size of each dog; therefore, the use of dilators and cannulas of fixed size that are used in human medicine is expected to be reduced.

There were also limitations to the 3DPSSG in this study. First, there were a limited number of cases and no control group. Further prospective comparative studies are needed to evaluate the benefits of this guide relative to other MISS for MI-MHC. Second, the production of guides requires CT imaging, which requires additional anaesthesia, time for designing and producing guides, and funding. Third, learning curves are necessary because the surgeons may not be familiar with the MISS approach and the instruments used [25]. Fourth, stereolithographically-printed surgical guides may have errors compared to the intended design, and a previous study reported that such errors may occur in up to 13% of guides used for dental surgery [31]. Printing processes, such as object build orientation and positioning, and post-processing, such as curing and sterilisation, can also modify the guide [32]. Fifth, since this study was conducted using cadavers, it was not possible to evaluate various conditions that may occur in live dogs. It is difficult to objectively evaluate whether major structures are damaged when using the MISS approach. In addition, the postoperative neurological status and bleeding that may occur during the drilling process could be accurately evaluated. Finally, this guide is not suitable for treating extensive lesions that are spread over a large area (e.g. Funkquist type 3 disc extrusions or epidural haemorrhage) [30, 33] or for creating a slot of the correct depth and height during the corpectomy procedure; therefore, it is necessary to design a different type of guide through further research.

## Conclusion

The 3DPSSG for MI-MHC used in this study is accurate and safe in cadaveric dogs, and it can be an alternative to the standard MHC or MI-MHC procedure. Using this guide, it was possible to perform the spinal cord decompression step of the MI-MHC procedure effectively, with magnification and illumination assisted by an endoscope. However, clinical applications are needed to evaluate this new technique, and further studies are required to overcome the limitations revealed in this study.

## Methods

### Cadaveric specimens and groups

Fifteen canine cadavers of different breeds weighing 3.1–34.4 kg was included. The dogs were euthanized for reasons unrelated to the present study. Prospective consent for the use of the dogs in the present study was obtained from the owners before use. All cadavers were stored at -20℃, and when thawed for research, they were stored at -4℃ for 72 h before the surgical procedure.

To evaluate the accuracy of the guide, randomisation software (http://www.random.org/) was used to determine on which side the surgery would be performed, which bone of the two lumbar vertebrae (L1 or L2) will insert the screw first, and which of the two surgeons will insert the screw first. Additionally, it was determined which surgeon would perform the MI-MHC procedure on which dog and how many times. The screws inserted by an experienced surgeon (JSK, a surgeon with seven years of experience) were classified as Group A (*n* = 15), and those inserted by an inexperienced surgeon (SEL, a surgeon with two years of experience) were classified as Group B (*n* = 15). The MI-MHC procedures performed by the experienced surgeon were classified as Group C (*n* = 8), those performed by the inexperienced surgeon were classified as Group D (*n* = 7). Seven dogs underwent a surgical procedure on the left side and eight dogs underwent a surgical procedure on the right side.

### Production of the surgical guides

Computed tomography (CT) images of the thoracolumbar vertebral columns of the dog cadavers were acquired (1-mm slice thickness; Toshiba Alexion 16; Toshiba Medical System). The resulting Digital Imaging and Communications in Medicine (DICOM) images were exported into medical image software (3D slicer, National Alliance for Medical Image Computing, Boston, MA, USA) for bone model creation. Stereolithography files were exported to computer-aided design software (Fusion 360, Autodesk, San Rafael, CA), and virtual 3D models of the thoracolumbar vertebrae column were created. A virtual surgical guide is created for each specimen. For the unity of the experiment, all surgical guides were manufactured for mini-hemilaminectomy-corpectomy (MHC) of the intervertebral disc between L1–L2. The 3D surgical guide was designed with a surgical part and a tube part to fix the guide to the vertebral body.

The central axis of the drilling and screw placement was designed based on the insertion angle of the bicortical spinal implant. For L1 and L6 vertebrae, it is recommended that the insertion angle of the spinal implant from vertical be 55° to 65° [34]. However, an evaluation based on the CT image of an individual patient is more accurate. Therefore, in this study, the insertion angle was set between 55° to 65°, depending on the shape of the specimen's vertebrae. To prevent the screw from penetrating into the spinal canal, the path of the screw was set to have a distance of 2 mm or more from the spinal canal based on preoperative CT images (Fig. 2A).

**Fig. 1:**
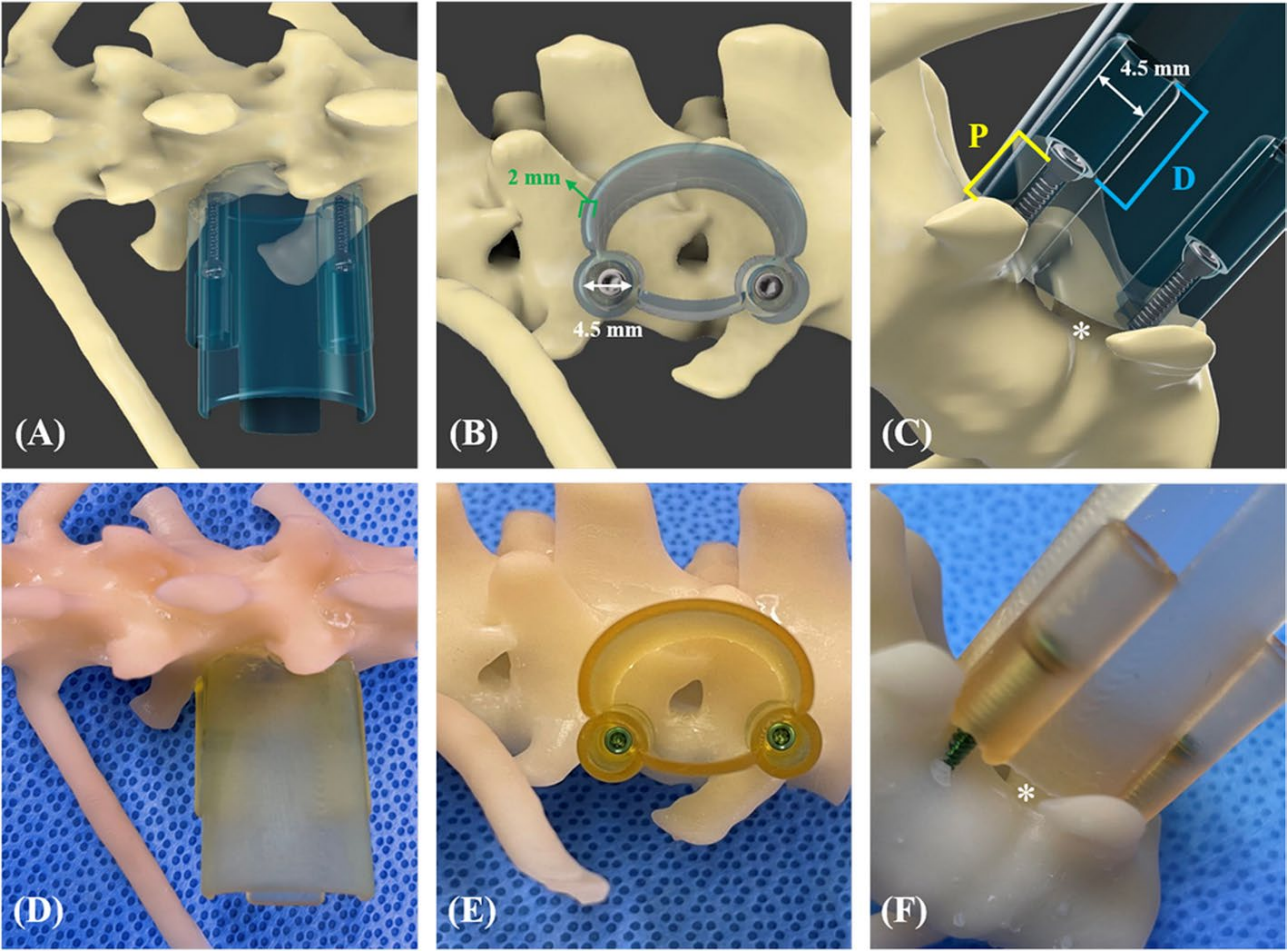
**A** to **C** Detailed components and design process of the 3DPSSG of MI-MHC. **D** to **F** The surgical guide was placed on the 3D printed biomodel of vertebrae to subjectively assess fit. (Fusion 360 was used to create 3D rendering images) *, the space for the nerve root and surrounding tissues; D, distal part of tube; P, proximal part of tube

To create patient-specific surgical guides, inverted virtual representation of the periforaminal area (composed of the dorsolateral part of the intervertebral disc, the accessory process, the cranial part of the pedicle of the caudal vertebra, and the caudal part of the pedicle of the cranial vertebra) between L1 and L2 was used to create the base of the surgical guide, which would fit onto the cortex in a unique position (Fig. 1A, D). For the area where the surgical part contacted the periforaminal area, the width and length were set according to the MHC, and the thickness of the guide wall was designed to be 2 mm for the stability of the guide (Fig. 1B, E). To prevent the surrounding soft tissue or skin from entering the guide and interfering with the surgical window, the height of the guide was set to be 1.5 cm higher than the skin of the incision area based on preoperative CT images.

The surgical part was designed to be oval in shape to minimise tissue damage and the incision size. The space was created on the ventral side of the guide so that it was not completely compressed by the vertebral body and so that the nerve root and surrounding blood vessels were not compressed (Fig. 1C, F).

**Fig. 2:**
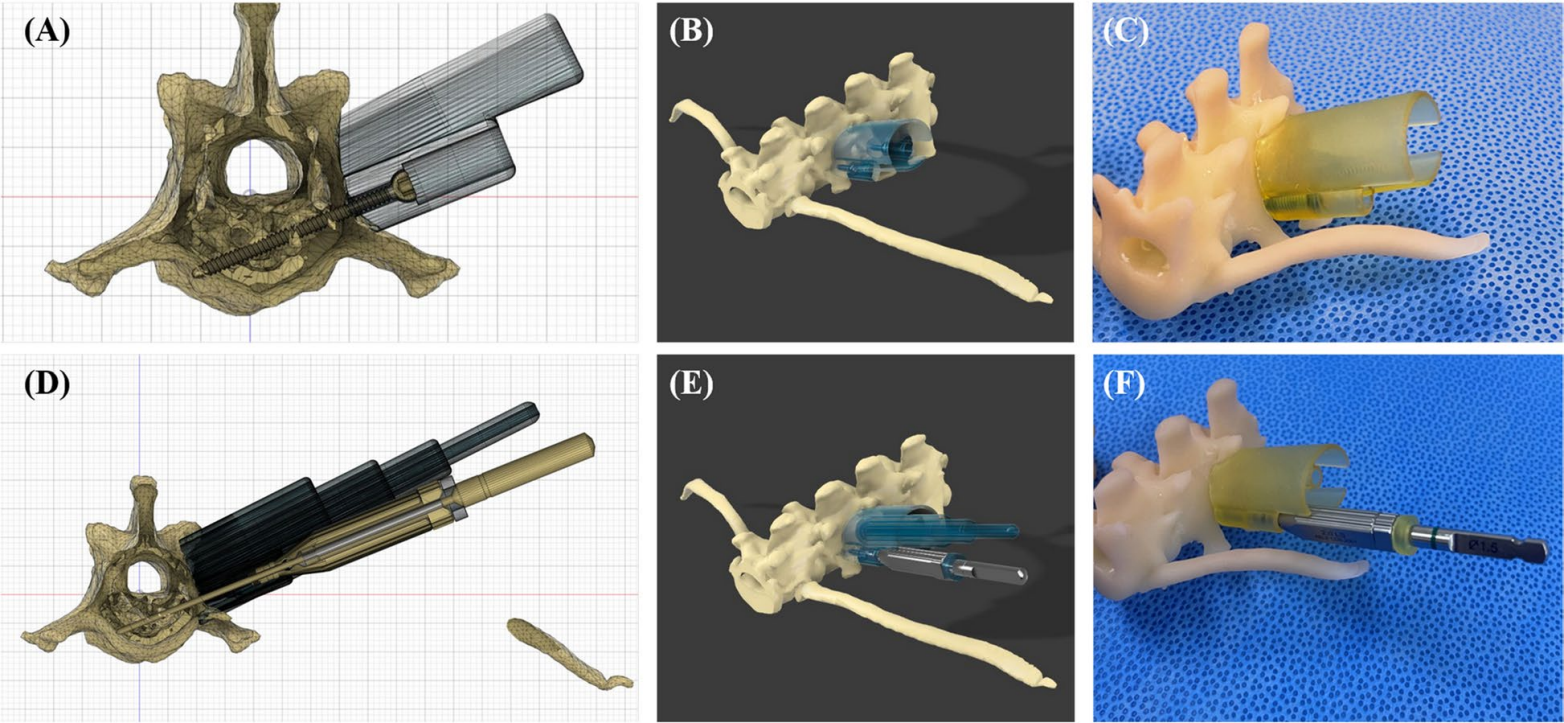
**A, B, D, E** Detailed components and design process of the 3DPSSG of MI-MHC. **C, F** The surgical guide and drill stopper were placed on the 3D printed biomodel of vertebrae to subjectively assess fit. d, minimum distance between screw and spinal canal

The tube was attached to the cranial and caudal sides of the surgical site. The tubes were designed with an inner diameter of 4.5 mm in the distal part to secure a space for the screwdriver to enter, and the thickness was set as 2 mm for stability (Fig. 1B, C). In order to compress and fix the guide with the vertebral body, it was planned to apply 1.5 / 2.0 mm cortical screw inside the tube in the proximal part in a lag fashion (Fig. 1C). Using the section analysis function in the software program (Fusion 360), the optimal length was measured such that the screw protruded with a length of 0.5 mm from the far cortex and was applied as a bicortical placement (Fig. 2A, B, C).

**Fig. 3:**
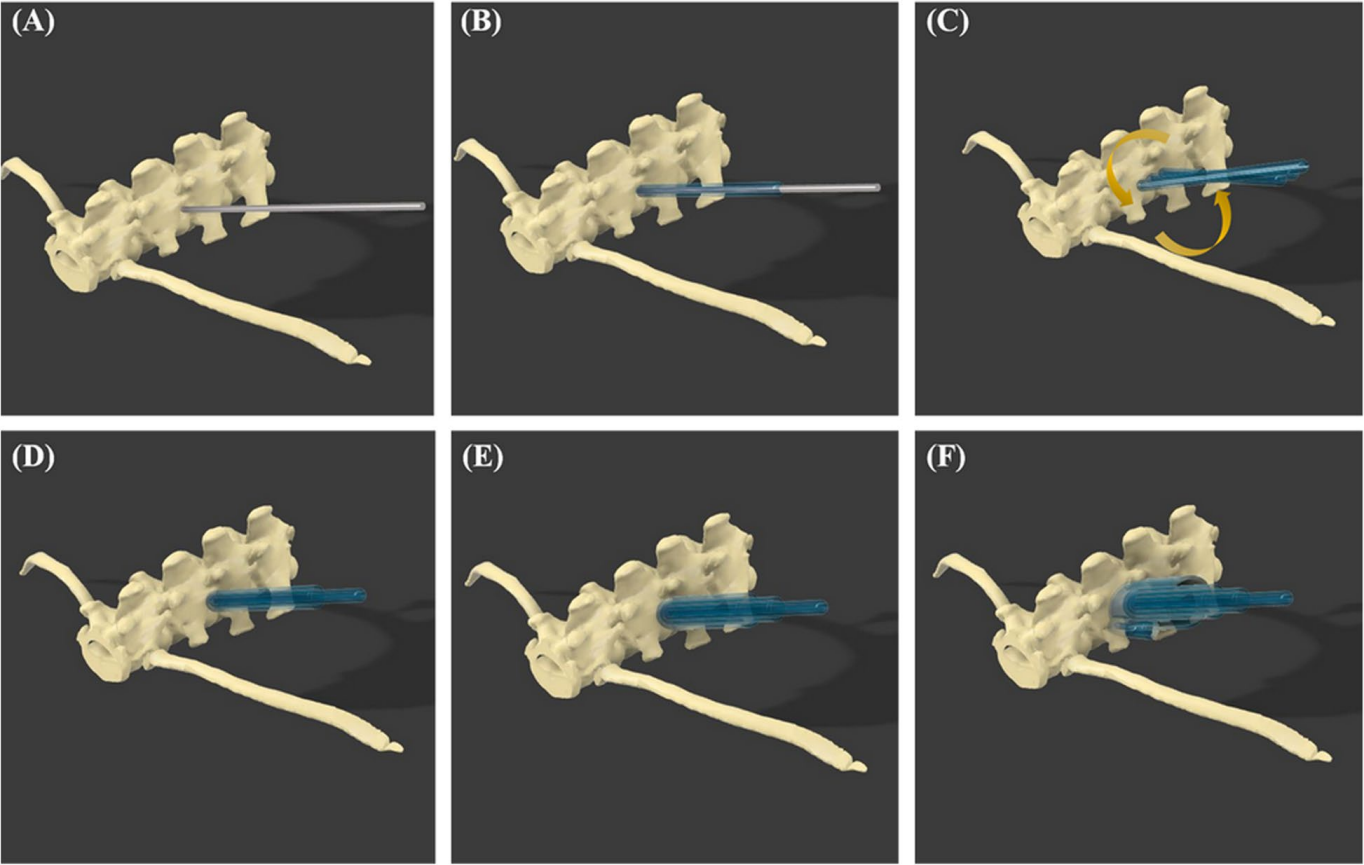
3D rendering images of the design and use of dilators for the MISS approach. Place the smallest dilator over the guide pin, insert larger dilators to expand between the soft tissues, install the guide, and remove the dilators

After placing the screw of the appropriate size, a hemispherical structure where the head of the screw could be seated and a structure that could function as a gliding hole were added. In the next process, drill stoppers were manufactured such that the drill bit protruded with a length of 0.5 mm from the far cortex (Fig. 2D, E, F). To implement the MISS approach in the process of installing the guide, dilators inspired by the tubular retractor system were designed (Fig. 3). The time consumed in designing a surgical guide to be applied to each specimen was measured.

Vertebral models were 3D-printed using a resin 3D-printer (A1, Zerone, Seoul, Korea), and a dental surgical guide resin (SG-100, Graphy, Seoul, Korea) was used. The surgical guides were 3D-printed using a resin 3D-printer (Pixel One, Zerone, Gyeonggi, Korea), and dental surgical guide resin (SG-100, Graphy, Seoul, Korea) was used. After printing, the guides were washed and dried for 30 min, and UV-light was cured at a wavelength of 405 nm for 60 min (3DP-100S, CUBICON, Gyeonggi, Korea).

### Cadaveric surgical procedure

**Fig. 4:**
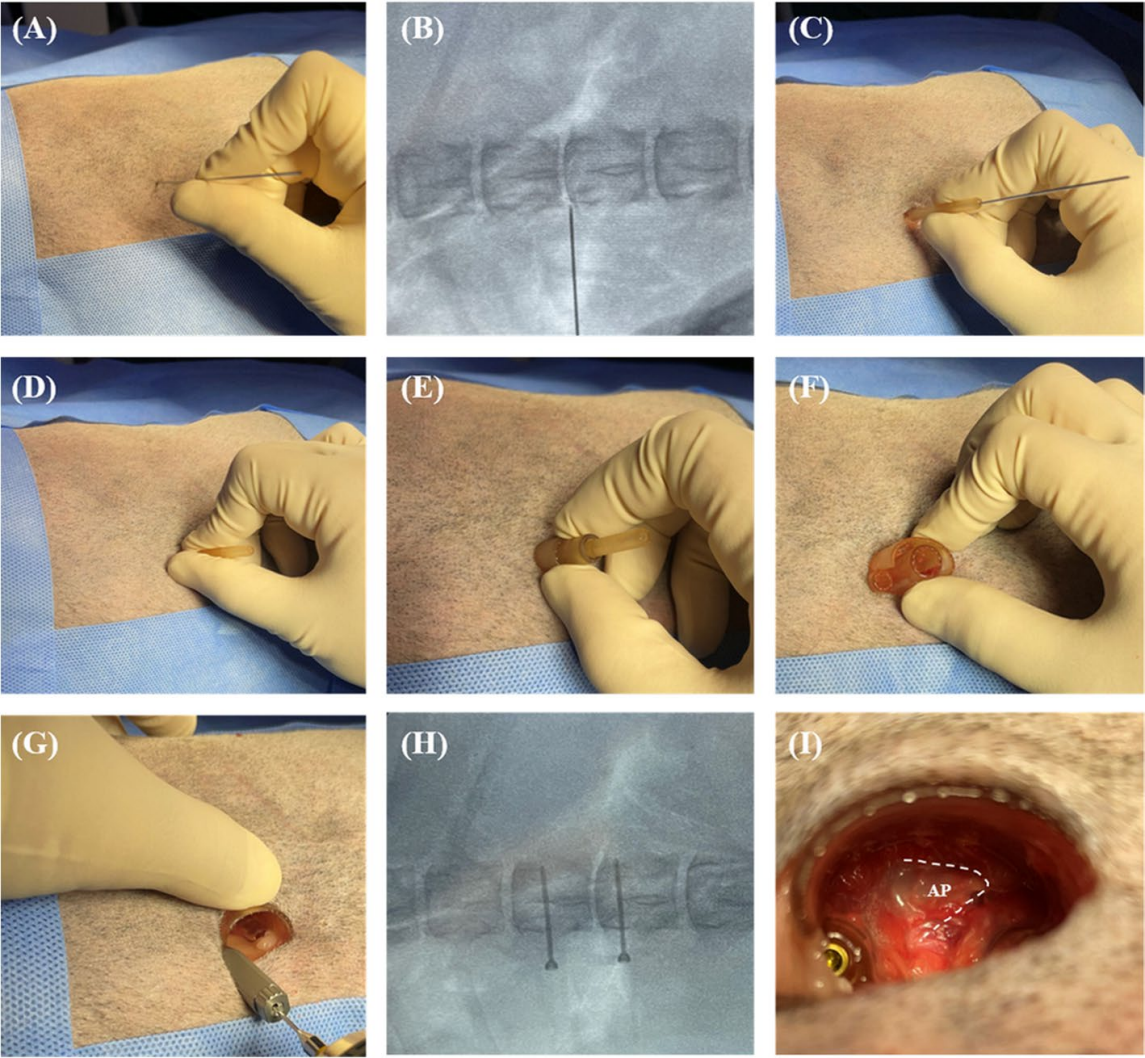
The process of surgical guide implantation. **A, B** insertion of guide pins into the disc space. **C** to **F** MISS approaches using dilators. **G** to **H** Fixation of surgical guide and vertebral body using screws and confirmation via intraoperative fluoroscopy. **I** Identification of periforaminal area through the guide's internal field. AP, Accessory process

All cadavers were positioned in 30° sternal oblique recumbency, with the planned side up on a tilting surgery table. A dorsolateral approach to the spine was performed at the planned site [35]. The position was maintained during the procedure by securing the cadaver using an immobilisation mattress (VACCUMAT, Genia, Saint-Hilaire de Chaléons, France). The MISS approach to the surgical site was performed by the leading author (JSK). After identification of the L1–L2 intervertebral disc space using intraoperative fluoroscopy (ZEN-2090 Pro, GENORAY Inc., Gyeonggi, Korea), a Kirschner wire (K-wire) was inserted through the skin and fascial incision into the epaxial musculature. The direction was toward the L1–L2 articular processes, and the correct K-wire location was verified using intraoperative fluoroscopy (Fig. 4A, B). After the first dilator was placed over the guide pin and was in contact with the bone, the guide pin was removed. The space between the soft tissues was widened by inserting the second and third dilators (Fig. 4C, D, and E). The surgical guide was positioned over the dilators, then the dilators were removed (Fig. 4F). The correct positioning of the surgical guide over the planned bone surface was confirmed using intraoperative fluoroscopy. Additionally, it was confirmed that the guide was stable without significant deviation when a manual force was applied in the craniocaudal and mediolateral directions. The remainder of the muscle and soft tissue were dissected away from the bone with a periosteal elevator and micro-rongeur.

The surgical guide was held in place manually, a 1.5/2.0 drill sleeve (ABLE Inc., Jeonbuk, Korea) was placed into the tube part, and the vertebrae were drilled using a 1.5-mm drill bit (ABLE Inc.) with a stopper manufactured to create a hole length that can safely penetrate both cortices. Subsequently, a stainless-steel self-tapping 2.0 mm cortical screw (ABLE Inc.) was placed bicortically. As the head of the screw was seated on the hemispherical structure, the guide and vertebral body were compressed in a manner similar to the lag fashion (Fig. 4G). After placing screw in each tube, we checked whether the guide was fixed in the proper position using intraoperative fluoroscopy (Fig. 4H). After the remaining soft tissue in the surgical guide was carefully removed using a micro-rongeur, the periforaminal area was exposed through the guide's internal field (Fig. 4I).

**Fig. 5:**
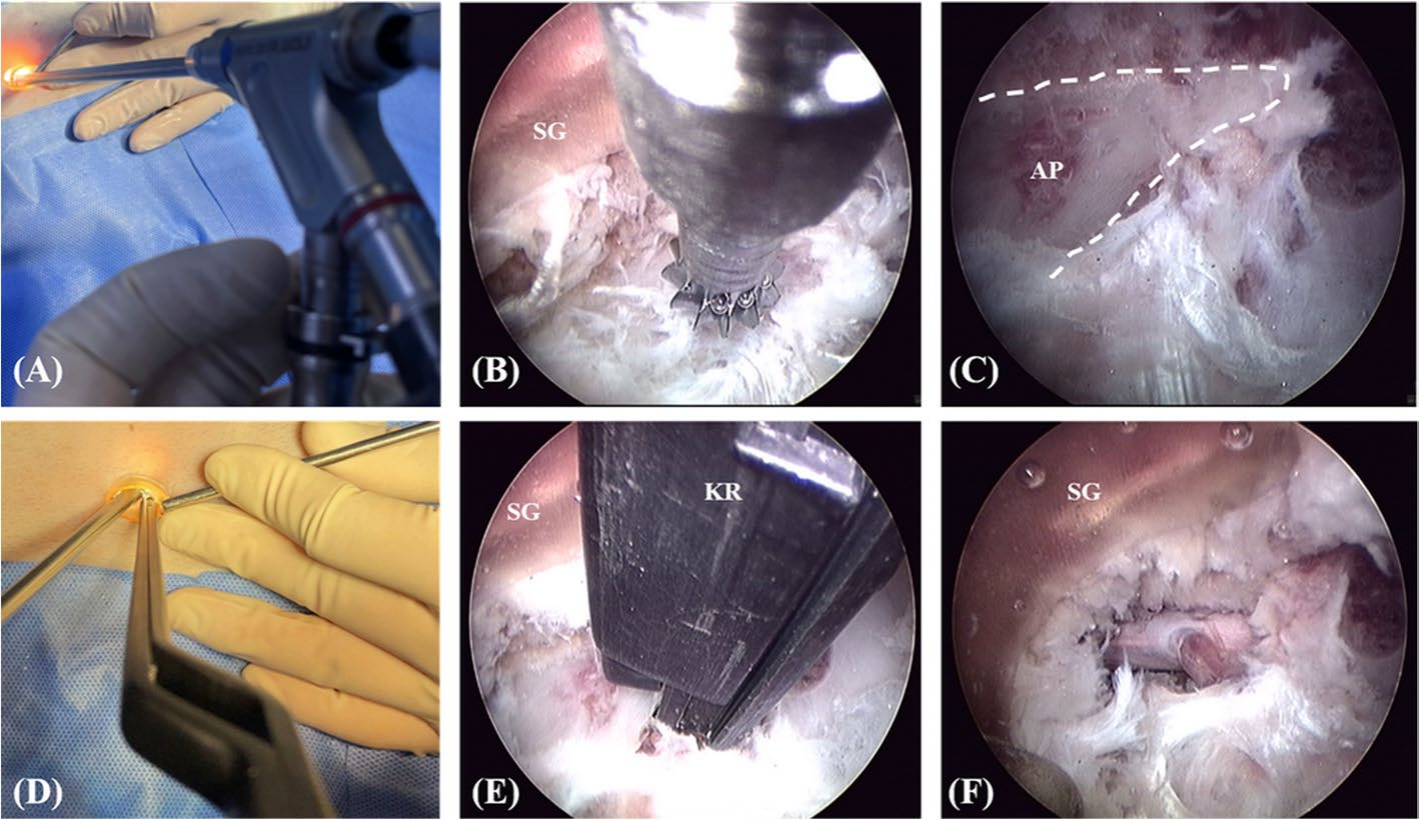
Surgical procedure. **A, B** Mini-hemilaminectomy was performed using high-speed surgical drill through intra-endoscopic working channel. **C** to **E** Removal of the accessory process and inner cortex using a 1 mm bayoneted Kerrison rongeur. **F** Intraoperative photograph of spinal cord and nerve root exposed. SG, surgical guide; AP, Accessory process; KR, Kerrison rongeur

**Fig. 6:**
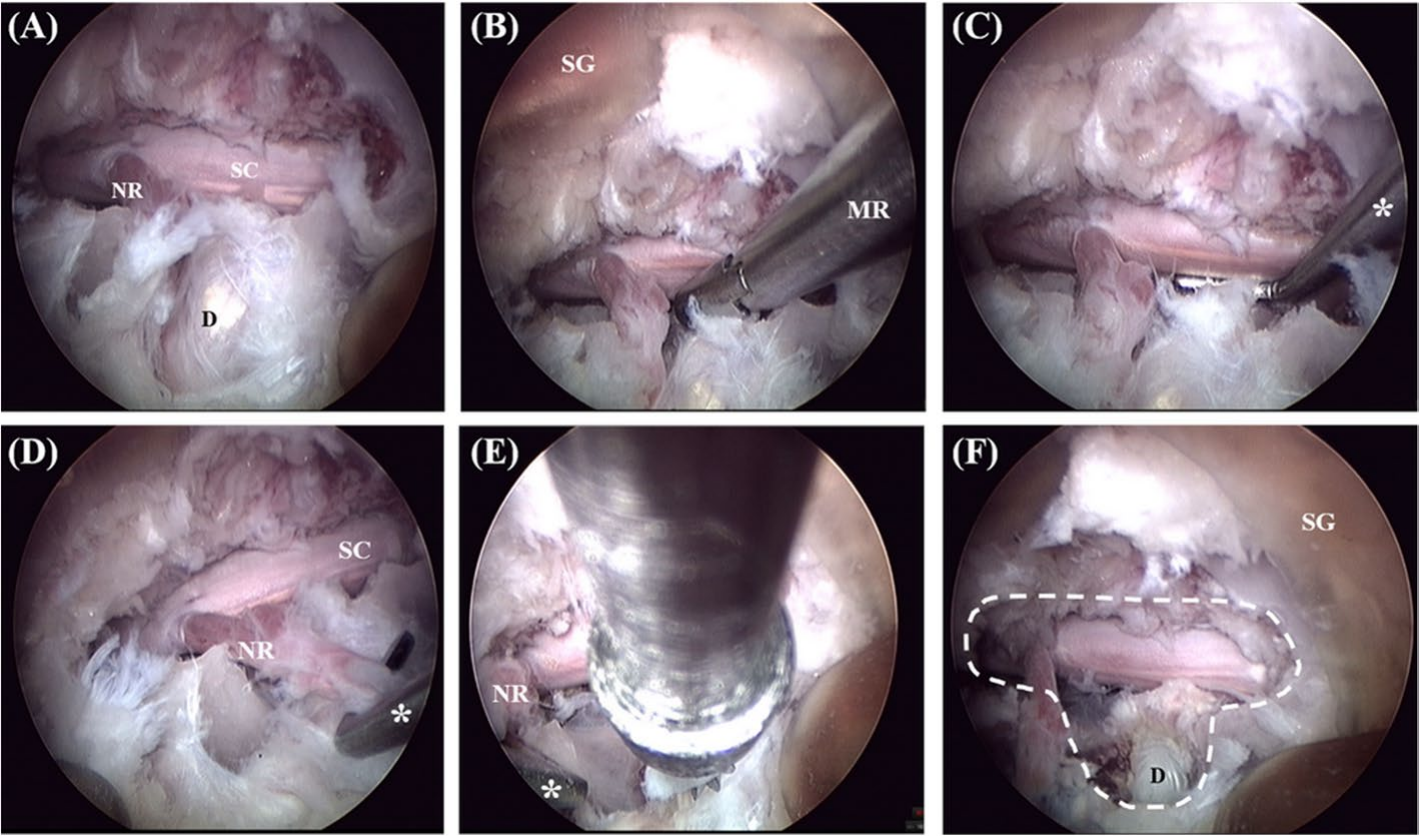
Surgical procedure (continued). **A** Intraoperative photograph after mini-hemilaminectomy. **B, C** Mimicking removal of disc material using nerve root retractor and micro-rongeur. **D, E** Corpectomy was performed using high-speed surgical drill with the nerve root retracted by the nerve root retractor. **F** Intraoperative photograph after MHC. SC, spinal cord; NR, nerve root; D, intervertebral disc; SG, surgical guide; *, nerve root retractor; MR, micro-rongeur

The remaining surgical procedures were performed using endoscopy-assisted microsurgery. The spinal endoscope (Richard Wolf GmbH, Knittlingen, Germany) had an outer diameter of 5.9 mm and usable length of 207 mm. It contained optics and a working channel with a diameter of 3 mm and a vision angle of 25°. First, the endoscope was introduced in the surgical guide, and the tendinous attachment of the longissimus muscle to the accessory process at the affected site was removed using the electrocautery and micro-rongeur (Richard Wolf GmbH, Knittlingen, Germany). A high-speed surgical drill with a 2.8-mm round carbide drill bit (Endospine bit, ABLE Inc.) was used to introduce the working channel of the endoscope and was controlled with a hand piece. Mini-hemilaminectomy was performed using a drill through the intra-endoscopic working channel with continuous irrigation using saline (0.9% NaCl) solution (Fig. 5A, B, C). To avoid interference from the surgical field of view, suction was mainly performed at the top of the guide, and if there was much bone debris generated during the drilling process, the suction tip was advanced deeper to improve visualisation (Fig. 5D). Drilling was performed through the outer cortex and the cancellous bone until the inner cortex was identified. A 1-mm bayoneted Kerrison rongeur and 1-mm bone curettes were used to remove the accessory process and inner cortex (Fig. 5E, F). The procedure was considered complete when the spinal cord was adequately visualised (Fig. 6A). After completion of the procedure, a micro-rongeur and nerve root retractor were used to mimic the removal of the disc material and examine underneath the spinal cord over the entire disc space (Fig. 6B, C).

For the next procedure, the dorsal and ventral spinal nerve roots were first bluntly dissected from their ligamentous attachments using a nerve root retractor and then displaced slightly cranially in order to gain an approach to the lateral aspect of the intervertebral disc. Beginning from the centre of the intervertebral disc, partial lateral corpectomy was performed in the cranial, caudal, and medial directions using a high-speed surgical drill (Fig. 6D, E). The partial lateral corpectomy slot was performed under the assumption that the disc material was concentrated unilaterally on the planned side (Fig. 6F). Including the surgical guide installation, the total surgical time was measured from the skin incision to closure.

### Postoperative CT and 3D software analysis

**Fig. 7:**
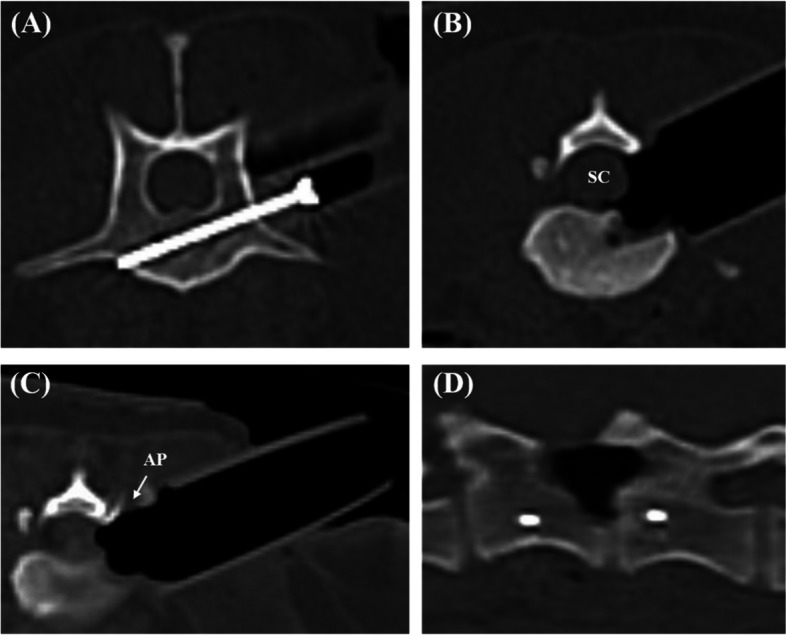
Postoperative CT images. **A** to **C** Transverse plane, **D** Sagittal plane. SC, Spinal cord; AP, Articular process

**Fig. 8:**
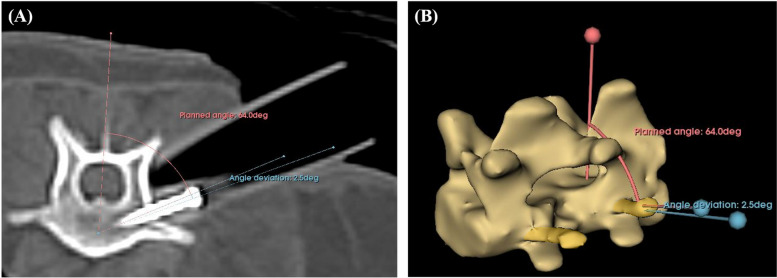
Analysis of screw angle deviation. In this specimen, the planned screw insertion angle was set to 64 degrees based on the spinous process (reference range 55 – 65 degrees), and the postoperative screw insertion angle was 66.5 degrees. The difference between the two angles (2.5 degrees) is the angle deviation. The yellow-colored area represents the screw

**Fig. 9:**
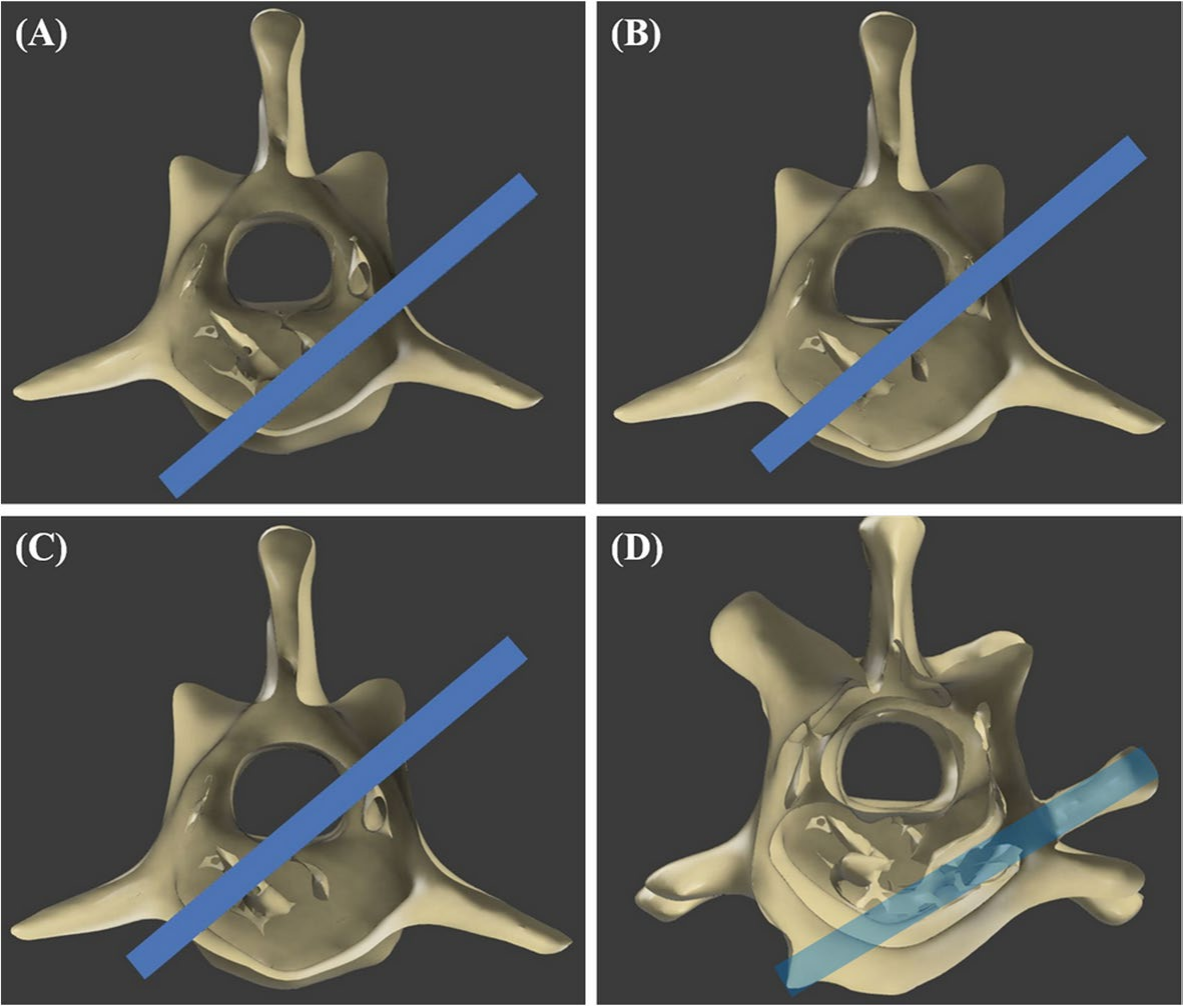
Images of modified Zdichavsky classification. **A** Grade I, optimally placed screw fully contained within the pedicle and vertebral body. **B** Grade IIa, partial penetration of the medial pedicle wall. **C** Grade IIb, full penetration of the medial pedicle wall (whole of screw diameter within vertebral canal). **D** 3D rendering images of a grade I screwed specimen. the transparent blue-colored area represents the screw

All the measurements were performed by a single investigator (JSK, DVM, PhD). The postoperative CT images (Fig. 7) were imported into the 3D planning software (3-D slicer, version 4.13.0) to analyse the accuracy of the screw placement in the surgical guide and the measurement of slot width and length. In the 3D planning software, the screw angle deviation between the intended screw trajectories and postoperative screw tracts was compared using angular measurements (Fig. 8). In addition, the length of the screw protruded from the far cortex was measured. The screw placement safety was subjectively graded using the modified Zdichavsky classification [29] (Fig. 9).

**Fig. 10:**
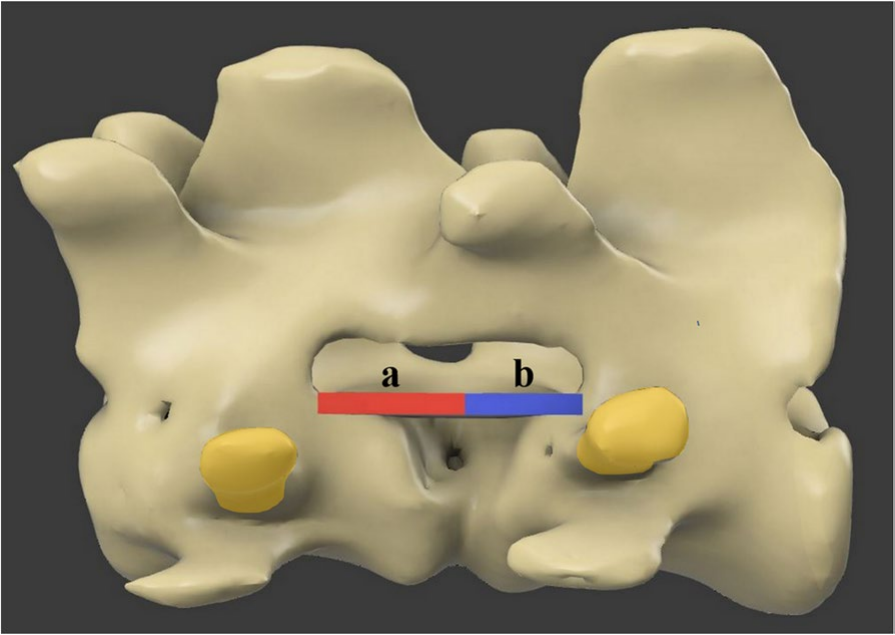
3D rendering images of the analysis for direction of bone window formation. The measurement for directional bias assessment was calculated as the cranial bone window length (**a**) divided by the caudal bone window length (**b**). The yellow-colored area represents the screw

To evaluate whether the bone window resulting from mini-hemilaminectomy was biased in the cranial or caudal direction, postoperative CT images were implemented as 3D rendering images using computer-aided design software (Fusion 360, Autodesk, San Rafael, CA). The length from the centre of the disc space to the end of the cranial direction and the end of the caudal direction was measured (Fig. 10). The directional bias assessment was calculated as the cranial bone window length divided by the caudal bone window length. If the value was greater than one, it was evaluated as biased in the cranial direction, and when the value was less than one, it was evaluated as biased in the caudal direction.

**Fig. 11:**
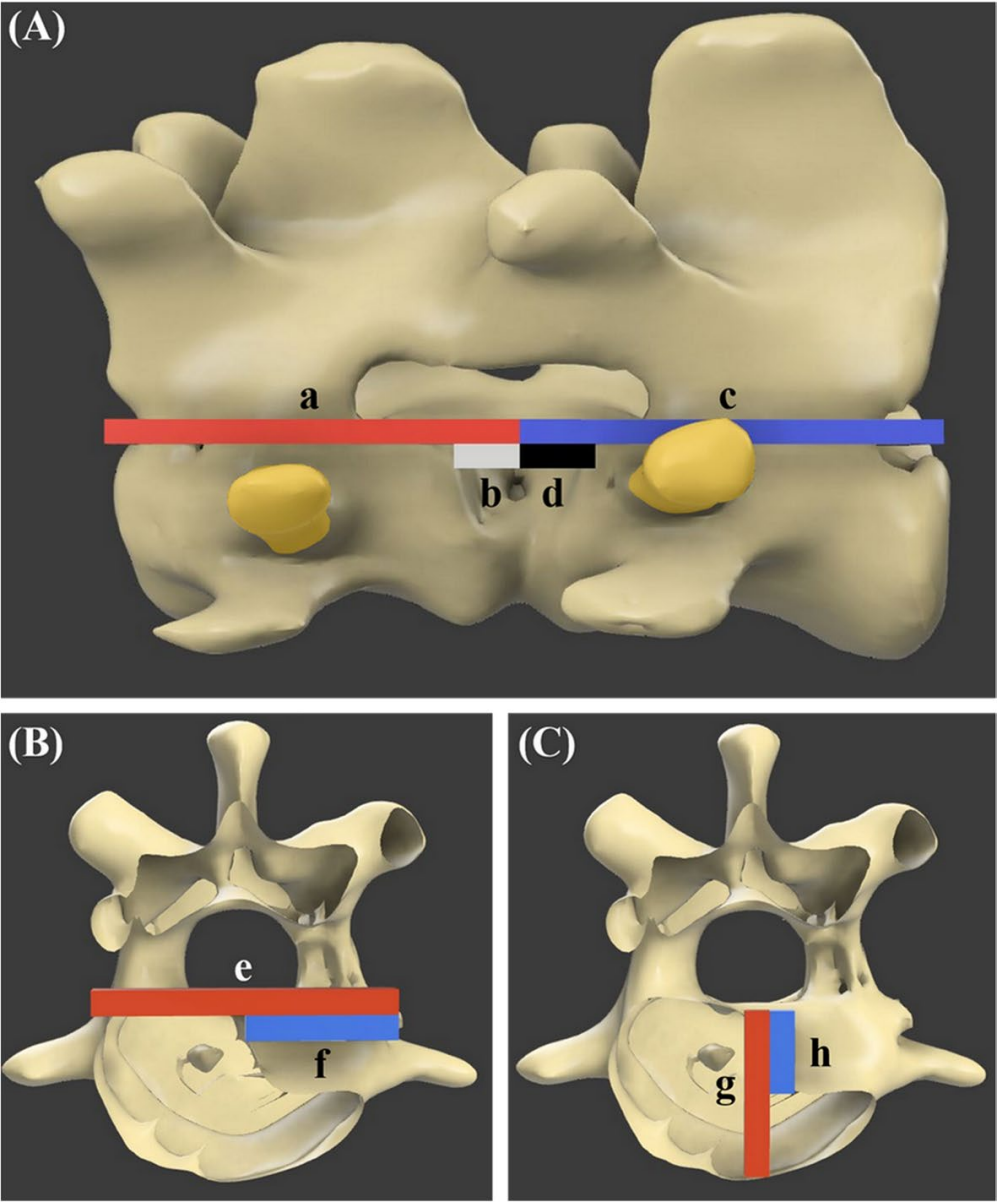
3D rendering images of the analysis of corpectomy slot length ratio (**A**), slot depth ratio (**B**), slot height ratio. The length ratio of the C3 part was calculated as b / a X 100, and the C4 part was calculated as d / c X 100. The depth ratio was calculated as f / e X 100 and the height ratio was h / g X 100 (The “/” sign means division and the “X” sign means multiplication). The yellow-colored area represents the screw

To evaluate the slot created by the surgical guide, using the section analysis function of the computer-aided design program (Fusion 360), the preoperative vertebral body length and the length of the bone resected in the lateral corpectomy process were measured in the sagittal plane of the 3D rendering images. The slot-length ratio was expressed as the ratio of the length of the resected bone to the preoperative vertebral length (Fig. 11A). The slot depth and height were measured in the transverse plane. The slot-depth ratio was expressed as the ratio of the slot depth to the preoperative vertebral body width (Fig. 11B). The slot-height ratio was expressed as the ratio of the slot height to the preoperative vertebral body height (Fig. 11C). These distances and ratios were calculated as percentages (%) using imaging software (Desktop ruler v3.8.6498; AVPSoft, Moscow, Russia).

### Data analysis

An a priori power analysis was not performed in this study. Descriptive statistics were acquired for body weight, guide design time, surgical time, screw angle deviation, length of the screw protruded from the far cortex, corpectomy slot-length ratio, corpectomy slot-depth ratio, and corpectomy slot-height ratio. Continuous data were tested for normality using the Kolmogorov–Smirnov test; they were found to be normally distributed and were reported as mean and standard deviation (SD). The Mann–Whitney test was used to compare values between experienced and inexperienced surgeons. Statistical significance was set at *p* < 0.05. Statistical analyses were conducted using SPSS (version 26.0; IBM, Armonk, NY, USA).

## Data Availability

The datasets used and/or analysed during the current study are available from the corresponding author on reasonable request.

## Abbreviations

IVDD: Intervertebral disc disease
3D: Three-dimensional
3DPSSG: Three-dimensional printed patient-specific surgical guide
MI-MHC: Minimally invasive mini-hemilaminectomy-corpectomy
MHC: Mini-hemilaminectomy-corpectomy
CT: Computed tomography
